# Genetic Determinants of the Association between Osteoarthritis and Psychiatric Disorders

**DOI:** 10.1155/2023/5253920

**Published:** 2023-08-03

**Authors:** Wenwen Chen, Jianwei Zhu, Xin Han, Yu Zeng, Can Hou, Yuanyuan Qu, Huazhen Yang, Yao Hu, Yajing Sun, Huan Song

**Affiliations:** ^1^Mental Health Center and West China Biomedical Big Data Center, West China Hospital, Sichuan University, Chengdu, China; ^2^Med-X Center for Informatics, Sichuan University, Chengdu, China; ^3^Department of Orthopedics and Orthopedic Research Institute, West China Hospital, Sichuan University, Chengdu, China; ^4^West China Biomedical Big Data Center, West China Hospital, Sichuan University, Chengdu, China; ^5^Center of Public Health Sciences, Faculty of Medicine, University of Iceland, Reykjavík, Iceland

## Abstract

**Background:**

The associations between hip/knee osteoarthritis (OA) and various psychiatry disorders, as well as the underlying genetic determinants, remain unclear.

**Methods:**

Based on the community-based prospective data of UK Biobank, we constructed two matched cohorts to assess the bidirectional associations between OA and five common psychiatry disorders. Then, we further examined the existence of overall genetic association for those disease pairs with demonstrated phenotypic association through polygenic risk score (PRS) prediction using individual-level genotyping data of UK Biobank and LD score regression (LDSC) analysis utilizing publicly available GWAS summary statistics. Last, also based on GWAS summary statistics, we performed enrichment analyses to pinpoint specific genetic determinants that might contribute to the observed overall genetic shares.

**Results:**

The phenotypic analyses revealed an elevated risk of hip/knee OA among individuals with any psychiatric disorders, compared to their matched unexposed individuals (hazard ratio (HR) = 1.62, 95% confidence interval (CI): 1.57-1.68), and vice versa (HR =1.93, 95% CI: 1.83-2.04). We further observed positive associations of knee OA with depression and stress-related disorder in the PRS analyses, which corroborated with the results of LDSC analyses (*r* for genetic = 0.20 (95% CI: 0.15-0.25) and *r* for genetic = 0.29 (95% CI: 0.19-0.40), respectively). Using GWAS summary statistics, we identified several shared genes and pathways, particularly the biological process related to HDAC histones, between knee OA and depression/stress-related disorder.

**Conclusions:**

Our study demonstrated a bidirectional association between OA and multiple psychiatric disorders, and the findings of shared genetic architectures between knee OA and depression/stress-related disorder provided possible targets for further mechanistic exploration and intervention development.

## 1. Introduction

Accumulating evidence from epidemiological studies supports a coaggregation of osteoarthritis (OA) and psychiatric disorders (e.g., depression, anxiety, and stress-related disorders), particularly in the aging population [1–4]. For instance, our prior efforts of visualizing disease trajectories identified OA as one of key diseases after a diagnosis of depression, featured by its shared common etiology with other chronic inflammation [5]. Likewise, psychiatric disorders have been associated with increased risks of a wide range of chronic physical conditions, including arthritis and chronic pain [6]. On the other side of the coin, both psychiatric symptoms (reported prevalence ranged from 20% to 58% [7, 8]) and diagnosis of psychiatric disorders (e.g., depression [9, 10] and anxiety [11, 12]) were common among OA patients. Furthermore, studies have shown that OA patients with comorbid psychiatric abnormalities might experience worsen symptoms [13], increased functional limitation [13], lower quality of life [14, 15], and prolonged hospitalization [16]. Also, they were reported to have worse functional recovery after arthroplasty [17], underscoring the importance of developing psychiatric interventions among OA patients.

The mechanisms for the bidirectional associations between OA and psychiatric disorders remain unclear. Together with persistent pain [18], behavior-related factors, such as reduced physical activity and movement restriction which could result in social isolation [19] and weight gain [20], might have contributed to the elevated incidence of psychiatric disorders among OA patients. In addition, the overactivated inflammatory processes (such as IL-1, IL-6, IL-15, and TNF*α*) that observed among both OA patients and patients with psychiatric disorders [21, 22] implied the possibility of shared biological pathways between these two traits. Such a notion gains further support from the longitudinal study indicating links between a broad range of psychiatric disorders and a higher disease susceptibility to OA [23]. However, although the genetic profiles of both OA and psychiatric disorders become clearer, benefiting from recent genome-wide association studies (GWAS) [24–27] with increasing number of involved cases, investigations exploring the shared genetic components of the phenotypic association were scarce. The existing ones merely focused on depression and overlooked the phenotypic and genetic dissimilarities between hip and knee OA [28, 29]. Thus, comprehensive assessments on the associations between different subtypes of OA and multiple psychiatric disorders, as well as their genetic shares, are of great importance, in terms of providing deep insights on the biological mechanisms underlying comorbid psychiatric disorders and OA and optimizing strategies for early identification and effective interventions.

Taking advantage of enriched data in UK Biobank, we aimed to perform a comprehensive assessment on the associations between OA and psychiatric disorders, from both phenotypic and genetic levels. Furthermore, we also planned to elucidate the possible genes and pathways that conferring risk of having both ends of diseases, which could be potential targets for further intervention studies, using publicly available GWAS summary statistics.

## 2. Methods

### 2.1. Study Design

**Figure 1:**
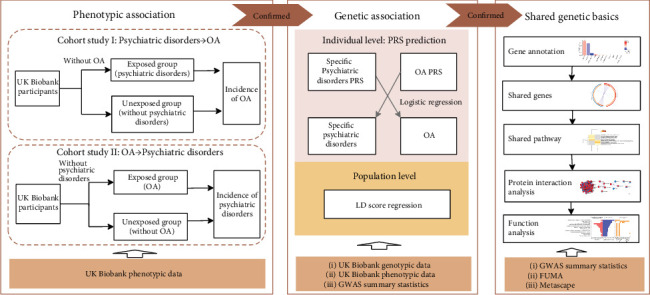
Study design. OA: osteoarthritis; PRS: polygenic risk score; GWAS: genome-wide association study.

Based on data from the large prospective cohort of UK Biobank and publicly available GWAS summary statistics, we explored the association between OA and psychiatric disorders, as well as their shared genetic components through three steps (Figure 1). First, among the UK Biobank participants, we assessed the bidirectional phenotypic association between OA and psychiatric disorders using two matched cohorts. The target psychiatric disorders included depression, anxiety, stress-related disorders, substance misuse, and psychotic disorders; and we performed the analysis first for hip/knee OA together, then for hip and knee OA separately. Second, for the disease pairs with verified phenotypic association in step one, we examined the existence of shared genetic background using polygenic risk score (PRS) prediction [30] and LD score regression (LDSC) [31]. At last, as the shared genetic basis from the aspect of overall genetic architectures has been detected, we further explored the possible specific genetic components (i.e., gene, pathway, and protein-protein interaction (PPI) networks) that contributed to the observed association. Detailed information about data sources and analytic strategies is presented in Supplementary Methods and Supplementary Table 1.

### 2.2. Phenotypic Association Analyses in UK Biobank

First, we performed a matched cohort study (cohort study I) to assess the association between psychiatric disorders and the subsequent risk of OA (i.e., psychiatric disorders→OA). After exclusion of participants who withdrew or had a history of any OA before psychiatric disorders, we included individuals with a psychiatric disorder diagnosis between 1997 and 2020 in the exposed group and psychiatric disorder free (i.e., without any of the five studied psychiatric disorders) individuals that 5-to-1 individually matched to the index patient by birth year (±2) and same sex to the unexposed group (Supplementary Figure 1). We followed up all participants from the index date until the first diagnosis of OA, death, or the end of study (Jan 1st, 2020), whichever occurred first. The psychiatric disorders and OA were ascertained by diagnoses documented in the UK Biobank inpatient hospital and primary care data, using the International Classification of Diseases (ICD) and READ v2/v3 codes (Supplementary Table 2). The overall median diagnostic accuracy was reported to be 80.3% (interquartile range, 63.3-94.1%) against reviewing of case notes [32]. For psychiatric disorders and OA specifically, the median positive predictive value was estimated to be 75% and 79%, respectively, in previous studies [33, 34]. We used the Cox models to assess the relative risk of incident OA, using time after the index date as the underlying time scale. All Cox models stratified by matched factors (birth year and sex) and adjusted for several confounders (see Supplementary Methods). In addition to treat hip/knee OA as one group, we further conducted analyses for hip OA and knee OA separately. After considering all psychiatric disorder together, we did separate analyses for five subtypes. We also performed stratified analysis by the source of OA identification (i.e., inpatient and only primary care).

Second, we assessed the OA-associated risk of psychiatric disorder (i.e., OA→psychiatric disorders) through a similar matched cohort study design (cohort study II, Supplementary Figure 2). We ascertained participants diagnosed with OA as the exposed group and compiled an unexposed group including 5 individuals who were free of OA and individually matched on year of birth (±2) and same sex with the index person. We followed up these two groups to identify newly diagnosed psychiatric disorders and calculated the hazard ratios of psychiatric disorders with their 95% CIs in the stratified Cox regression comparing exposure to unexposed. All the models are adjusted for the same covariates as cohort study I.

### 2.3. Genetic Association Analysis

#### 2.3.1. PRS Analyses in UK Biobank Participants

For the disease pairs (i.e., OA and the specific subtype of psychiatry disorders) with bidirectional phenotypic association, we examined the existence of shared genetic components by PRS prediction analysis using individual-level genotyping data. The PRS, which derived by summing of all risk alleles weighted by the effect size of each variant using standard clumping+thresholding (C+T) approach [35], represents an individual's overall genetic risk for a given disease [30]. It can be used to predict the risk of developing a second disease and, thereby, illustrating the genetic association between a disease pair [30]. After a standard GWAS quality control (see Supplementary Methods and Supplementary Figure 3), 338,573 participants with 7,130,905 SNPs were included in the PRS analyses. The summary statistics of GWAS for OA and specific psychiatric disorders, excluding sample overlap with UK Biobank, were served as the independent data for SNP selection and risk allele weighting [24, 27, 36–39]. PLINK (version 1.9) was used for the PRS profiling.

In the validation step, we examined the associations between generated PRSs and the corresponding phenotypes in the UK Biobank using logistic regression, adjusting for sex, birth year, genotyping batch, and first ten PCs. The PRS that significantly associated with its corresponding phenotype (Supplementary Table 3-4) and having the highest Nagelkerke's *R* squared (i.e., indicating the largest variance explained, ranged between 1.27% and 4.98%) was used to measure the associations of interest (i.e., between PRSs for OA and risk of specific psychiatry disorder, and vice versa), presenting as odds ratios (ORs) with 95% CIs derived from logistic regression models adjusted for the same covariates mentioned above. These analyses were carried out with R software (version 4.0), and two-sided *p* < 0.05 was considered significant.

#### 2.3.2. LD Score Genetic Correlation Analysis

The degree of genetic correlation or pleiotropy between OA and depression at population level was additionally evaluated using cross-trait LD score regression based on aggregate-level genetic data (i.e., publicly available GWAS summary statistics of both traits, as described in data source and Supplementary table 2) [31]. In the LD score genetic correlation analyses, we computed the overall (i.e., genome-wide) correlation (i.e., *r* for genetic) between two phenotypes by comparing the effect estimates of two GWASs, which indicated the proportion of variance that two traits share due to common genetic factors. It is implemented in the LDSC (version 1.0.1) software, using SNP panel of European ancestry LD scores from 1000 genomes as reference [31].

### 2.4. Gene Mapping and Enrichment Analysis

For the disease pairs with both bidirectional phenotypic and genetic associations, we further identified their shared genetic components at level of genes, pathways, and PPI, using the web-based FUMA (http://fuma.ctglab.nl/) [40] and Metascape platforms (https://metascape.org/) [41].

Specifically, we used two core functions of FUMA: (1) the SNP2GENE process, in which we mapped SNPs (GWAS-p thresholds < 1 × 10^−6^ based on the provided GWAS summary statistics with the largest sample sizes [25, 26, 36, 38, 39]) to risk genes for OA and specific psychiatry disorder separately, using three mapping strategies (positional, expression quantitative trait loci, and chromatin interaction mapping), and identified the shared genes between them by merging the two sets of risk genes, and (2) the GENE2FUNC process, where gene set enrichment analysis was performed based on these shared genes in biological context (i.e., the GWAS Catalog) [42].

We also performed pathways and PPI enrichment analysis based on Metascape [41]. In brief, for multiple gene lists generated by FUMA, Metascape identified the top enriched pathways across the two gene lists by integration of more than 40 current biological databases (including GO process and KEGG pathway) using its default parameters [41]. Additionally, PPI enrichment analysis first formed highly complicated protein network for the input genes base on a set of databases (i.e., STRING and BioGRID), and then, extract densely connected protein complexes embedded in the large protein network using the Molecular Complex Detection (MCODE) algorithm [41]. The top three most significantly enriched ontology terms were combined to annotate putative biological roles for each MCODE complex.

## 3. Results

### 3.1. Bidirectional Phenotypic Association between OA and Psychiatric Disorders

The cohort study I involved a total of 303,258 individuals, including 50,543 exposed individuals with a diagnosis of any psychiatric disorders and their 252,715 matched unexposed individuals (Supplementary figure 2). The mean age of participants at index date was 52.9 years. During a mean follow-up of 12.3 years, we identified 21,677 hip/knee OA. After adjusting for multiple confounders, 62% elevated risk of hip/knee OA was observed among individuals with any psychiatric disorders, compared to their matched unexposed individuals (hazard ratio (HR) = 1.62, 95% confidence interval (CI): 1.57-1.68). The excess risk was more pronounced for knee OA (hip OA: HR = 1.22, 95% CI: 1.16-1.29; knee OA: HR = 1.88, 95% CI: 1.80-1.96). Similar risk patterns were observed for all studied subtypes of psychiatric disorders except for psychotic disorder (Table 1). By severity of psychiatric disorders, increased risks of OA were observed after psychiatric disorders diagnosed merely in primary care, as well as those that required inpatient care (Supplementary Table 5).

Similarly, with the purpose of assessing the risk of psychiatry disorders after OA, the cohort study II enrolled 268,518 individuals including 44,753 hip/knee OA and 223,765 matched unexposed individuals. The mean age at index date was 63.6 years. During a mean follow-up of 7.79 years, 7,363 individuals received a diagnosis of psychiatric disorders. After adjusting for multiple confounders, hip/knee OA patients experienced a 93% increase risk of any psychiatric disorders (HR = 1.93, 95% CI: 1.83-2.04) compared with matched OA free individuals. The excess risks were observed for all subtypes of psychiatric disorders, with the highest estimate for stress-related disorder (HR = 2.33, 95% CI: 2.03-2.68) and anxiety (HR = 2.11, 95% CI: 1.91-2.33) (Table 1). In the separate analysis by data source, we observed more pronounced risk of psychiatric disorders after OA diagnosed from primary care (HR = 3.49, 95% CI: 3.18-3.84) (Supplementary Table 6).

### 3.2. Genetic Association between Two Diseases

The genetic association analyses were performed for disease pairs with demonstrated bidirectional phenotypic associations, including depression, anxiety, stress-related disorder, and substance misuse, with hip/knee OA, respectively (Table 2). In the PRS prediction analysis, we observed bidirectional associations of knee OA with depression (depression PRS and knee OA: OR = 1.04, 95% CI: 1.03-1.05, *p* = 8.52^∗^10^−9^; and vice versa: OR = 1.04, 95% CI: 1.02-1.05, 1.97⁣^∗^10^−5^) and stress-related disorder (stress-related disorder PRS and knee OA: OR = 1.04, 95% CI: 1.03-1.06, *p* = 2.26^∗^10^−11^; vice versa: OR = 1.04, 95% CI: 1.01-1.06, *p* = 2.23^∗^10^−3^), but not with anxiety or substance misuse. Similar results were observed for hip/knee OA, whereas the analyses for the genetic association between hip OA and psychiatry disorders got null results (Table 2).

The results of LDSC corroborated the findings of PRS prediction analyses, indicating a genetic association between knee OA and depression (*r* for genetic = 0.20, 95% CI: 0.15-0.25, *p* = 1.75^∗^10^−15^, suggesting one-fifth of the observed association between these two phenotypes could be explained by shared genetic components), as well as between knee OA and stress-related disorder (*r* for genetic = 0.29, 95% CI: 0.19-0.40, *p* = 9.12^∗^10^−8^) (Table 3).

### 3.3. Identification of Shared Genes and Pathways

**Figure 2:**
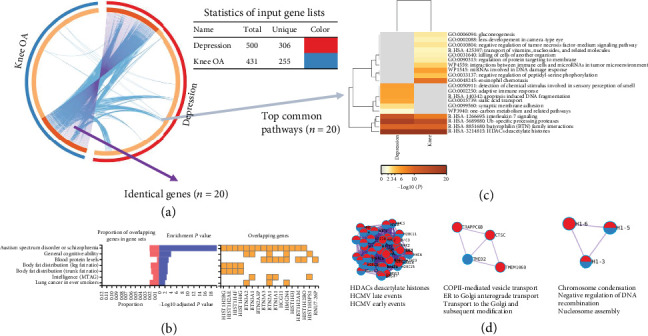
Identification of genes and pathways shared by knee osteoarthritis (OA) and depression. (a) Overlap between gene lists. Each candidate gene is assigned to one spot on the arc of the corresponding gene lists. Dark orange color represents the genes that appear in both lists, and light orange color represents genes that are unique to that gene list. Purple curves link identical genes, and blue curves link genes that have different identities but share an enriched pathway/process (i.e., they represent the functional overlaps among gene lists). (b) The functional relevance of the identified genes, based on reported genes from the GWAS Catalog. The shared genes (*n* = 20) were enriched for representation in various diseases or trait-related gene sets using the GENE2FUNC of the FUMA tool. (c) Heatmap of enriched pathways across input gene lists, colored by *p* values, one row per pathway, using a discrete color scale to represent statistical significance. Gray color indicates a lack of significance. (d) Shared MCODE component identified in protein-protein interaction network and the top pathways of the corresponding components, where each node represents a protein with a pie chart encoding its origin (i.e., blue for knee OA and red for depression).

We made further efforts on the identification of shared genes and pathways for the two disease pairs with observed phenotypic and genetic association (i.e., knee OA and depression and knee OA and stress-related disorder). For the disease pair of knee OA and depression, we first mapped 431 and 3365 risk genes for knee OA and for depression, respectively, based on SNPs with GWAS-*p* < 1^∗^10^−6^. Then, to avoid detecting signals unduly driven by one side (i.e., due to the imbalanced number of risk genes), we used the top 500 risk genes for depression and all risk gene (*n* = 431) for knee OA for shared gene identification, which resulted in 20 identified genes (Figure 2(a) and Supplementary Table 7). According to GWAS Catalog, those genes were functionally relevant to mental disease and body fat distribution (Figure 2(b)). Further pathway analysis using Metascape identified four common pathways, including interleukin-7 (IL-7) signaling, Ub-specific processing proteases, butyrophilin (BTN) family interactions, and HDAC deacetylate histone (Figure 2(c)). Moreover, the HDACs deacetylate histones pathway was also observed in PPI network analysis (Figure 2(d)).

**Figure 3:**
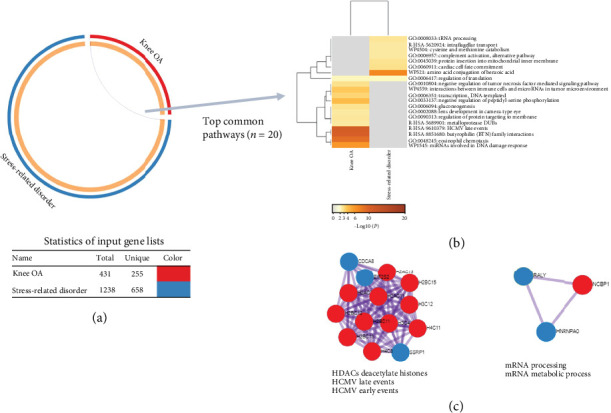
Identification of genes and pathways shared by knee osteoarthritis (OA) and stress-related disorder. (a) Overlap between gene lists. Each candidate gene is assigned to one spot on the arc of the corresponding gene lists. Dark orange color represents the genes that appear in both lists, and light orange color represents genes that are unique to that gene list. Purple curves link identical genes, and blue curves link genes that have different identities but share an enriched pathway/process (i.e., they represent the functional overlaps among gene lists). (b) Heatmap of enriched pathways across input gene lists, colored by *p* values, one row per pathway, using a discrete color scale to represent statistical significance. Gray color indicates a lack of significance. (c) Shared MCODE component identified in protein-protein interaction network and the top pathways of the corresponding components, where each node represents a protein with a pie chart encoding its origin (i.e., red for knee OA and blue for stress-related disorder).

For the disease pair of knee OA and stress-related disorder, we identified one gene shared by risk gene sets for both traits (Figure 3(a)), which was however not functionally relevant to any disease according to GWAS Catalog. In the pathway analysis, we detected one significant pathway regarding regulation of translation (Figure 3(b)). The PPI network analysis detected two MCODE components, which were relevant to pathway of HDACs deacetylate histones and mRNA metabolic (Figure 3(c)).

## 4. Discussion

Utilizing enriched phenotypic and genetic data in UK Biobank, as well as the publicly available GWAS summary statistics, our study demonstrated the link between different types of OA and multiple psychiatry disorders from phenotypic to genetic levels. Specifically, the results of community-based, longitudinal cohort studies of more than 200,000 participants confirmed the bidirectional association between OA and psychiatry disorders. We further verified the genetic associations between knee OA and two specific psychiatry disorders (i.e., depression and stress-related disorder), based on PRS prediction analyses and LDSC analysis. In addition, we identified a series of shared genes and biological pathways between knee OA and depression/stress-related disorder, which could shed a light on understanding of the common pathogenesis between these two diseases.

To the best of our knowledge, this is the first large cohort study that thoroughly discussed the connection between hip/knee OA and psychiatry disorders from both directions, at phenotypic and genetic level. Previous epidemiological studies have revealed increased risk of psychiatric disorders (e.g., depression, anxiety, and stress-related disorders) among OA patients [2–4], whilst evidence regarding the impact of psychiatric disorders on OA is relatively limited. Only one longitudinal study of approximately 300,000 participants, based on data of health insurance register, reported a 44% increased odds (95% CI: 39-49%) of developing OA after a diagnosis of psychiatric disorders (i.e., affective psychoses, personality disorders, and alcohol and drug dependence or abuse) during a 7-year follow-up period [23]. The findings of present study collaborated with previous epidemiological studies, indicating a bidirectional association between OA and psychiatric disorders.

The detailed mechanisms between hip/knee OA and psychiatry disorders remained unclear, although several potential explanations have been proposed previously. For instance, the persistent pain [18], reduced physical activity and movement restriction [43], might contribute to the elevated incidence of psychiatric disorders among OA patients. Additionally, shared etiologies were also a rational speculation, since overactivated inflammatory factors (such as IL-1, IL-6, IL-15, and TNF*α*) and altered immune response were observed among both OA and psychiatric disorders patients [21, 22, 44]. Indeed, recent cross-trait genetic studies revealed that shared genomic loci between major depression and OA, particularly those enriched in the “mechanosensory behaviors” pathway, may partially explain such an association [28, 29]. This notion gained further support from a disease trajectory study, where OA was found as one of key diseases after depression and congregated with other inflammation-related conditions [5]. Also, abnormal immune reaction, manifested as increased risk of subsequent autoimmune diseases after stress-related disorder, has been reported in a large-scale cohort study [45].

Adding to the existing literature, our functional enrichment analysis demonstrated the contribution of possible common pathways in the pathophysiological mechanism of comorbidity profiles between knee OA and depression, as well as stress-related disorders. And the identified biological pathways were mainly related to immune, inflammation, and histone modification biological process. It has been reported that IL-7 plays a role in the growth of murine B-cell precursors in bone marrow [46], and the BTN family modulates T-cell responses upon antigen presentation and mediates peripheral T-cell maintenance and proliferation [47]. One of the Ub-specific processing proteases (USP13) can ameliorate osteoarthritis by restraining oxidative stress and inflammation [48]. As the role of altered inflammatory and immune response in the development of psychiatric disorders was also revealed [22, 44], it is plausible that pathways involved in immune- and inflammation-related processes can act as potential links between OA and psychiatric disorders. In addition, the HDAC pathways, identified in both pathway analyses and PPI network analyses of our study, were considered important in the regulation of numerous histone modification-related biological processes in various diseases and have been validated as targets for drug design, for treating cancer and depression [49–51]. Previous studies also reported multiple roles of HDACs in the pathogenesis of OA interacting with cartilage and chondrocyte development [52, 53]. Taken together, the identification of those genetic genes and pathways can advance our understanding about the potential biological mechanisms underlying the observed associations and might aid development of effective prevention and treatment strategies for both diseases.

Our present study has several strengths. First is the combined use of multiple data sources, including UK Biobank phenotypic data, individual genetic data, and population level GWAS summary statistics, which prompted us to complete an atlas of association between OA and psychiatry disorders from phenotype to genetic basis. Second, our study thoroughly explored multiple web-based platforms and strategies during pathway analysis and obtained relatively consistent evidence for shared genetic basis underlying the phenotypic association.

However, several potential limitations should also be noted. First, the UK Biobank participants are not representative of the general UK population, given that it recruited only 5.5% of the target population, and the participants were predominately white [54, 55]. Also, the analyses of genetic basis between OA and psychiatry disorders were merely based on data of European populations, due to the lack of large-scale GWAS studies in other ethnic groups. Therefore, our results should be interpreted cautiously, and their generalizability to all UK population and other ethnic groups needs further investigations. Second, as only a few genetic loci were identified due to limited samples size for GWAS of stress-related disorders [36], our exploration on the shared genetic mechanisms between OA and stress-related disorders provides only suggestive evidence. At last, although we emphasized the consistent finding using different analysis strategies, the identified shared genetic components should be further examined in functional experimental studies.

In conclusion, our study demonstrated a bidirectional association between OA and multiple psychiatric disorders. Further analyses on shared genetic architectures between knee OA and depression/stress-related disorder identified several potential targets, such as biological processes involved in immune, inflammation, and histone regulation, which might be critical for further mechanistic exploration, as well as intervention development for disturbing the increased risk of knee OA among patients with depression/stress-related disorders, and vice versa.

## Figures and Tables

**Table 1:** Longitudinally bidirectional association between osteoarthritis (OA) and psychiatric disorders.

	Hip/knee OA		Hip OA		Knee OA	
	No. of cases (incidence^a^) in patients/matched individuals	HR (95% CI)^b^	No. of cases (incidence^a^) in patients/matched individuals	HR (95% CI)^b^	No. of cases (incidence^a^) in patients/matched individuals	HR (95% CI)^b^
*Association of psychiatric disorders with subsequent OA*						
Any psychiatric disorders	5342 (8.567)/16335 (5.281)	1.62 (1.57-1.68)	1816 (2.791)/7125 (2.259)	1.22 (1.16-1.29)	3865 (6.102)/10111 (3.23)	1.88 (1.80-1.96)
Depression	1702 (8.952)/4691 (4.986)	1.68 (1.58-1.78)	571 (2.871)/2080 (2.17)	1.27 (1.15-1.40)	1232 (6.368)/2885 (3.031)	1.90 (1.77-2.05)
Anxiety	1428 (8.471)/4466 (5.394)	1.60 (1.50-1.71)	469 (2.663)/1964 (2.327)	1.16 (1.04-1.29)	1059 (6.194)/2751 (3.283)	1.89 (1.75-2.04)
Stress-related disorder	796 (8.757)/2317 (5.157)	1.64 (1.50-1.79)	257 (2.702)/1018 (2.223)	1.18 (1.02-1.36)	587 (6.355)/1426 (3.138)	1.97 (1.77-2.19)
Substance misuse	1807 (8.309)/5771 (5.298)	1.69 (1.58-1.82)	642 (2.836)/2496 (2.247)	1.34 (1.20-1.50)	1280 (5.796)/3578 (3.246)	1.94 (1.78-2.11)
Psychotic disorder	33 (3.355)/223 (4.544)	0.87 (0.56-1.35)	10 (1)/103 (2.069)	0.68 (0.32-1.44)	25 (2.531)/131 (2.642)	0.98 (0.57-1.70)
*Association of OA with subsequent psychiatric disorders*						
Any psychiatric disorders	2091 (5.907)/5272 (3.034)	1.93 (1.83-2.04)	558 (4.337)/1846 (2.96)	1.43 (1.30-1.58)	1544 (6.836)/3438 (3.078)	2.23 (2.09-2.38)
Depression	702 (1.915)/1518 (0.8359)	2.07 (1.88-2.28)	193 (1.464)/509 (0.7799)	1.78 (1.50-2.12)	514 (2.185)/1011 (0.8667)	2.24 (1.99-2.51)
Anxiety	635 (1.729)/1461 (0.8046)	2.11 (1.91-2.33)	164 (1.24)/534 (0.8183)	1.50 (1.25-1.80)	474 (2.011)/930 (0.7972)	2.48 (2.20-2.79)
Stress-related disorder	340 (0.9198)/663 (0.3651)	2.33 (2.03-2.68)	88 (0.6624)/231 (0.354)	1.75 (1.35-2.27)	255 (1.074)/432 (0.3703)	2.71 (2.29-3.19)
Substance misuse	719 (1.961)/1915 (1.055)	1.83 (1.65-2.04)	189 (1.431)/691 (1.059)	1.23 (1.01-1.50)	533 (2.266)/1232 (1.056)	2.25 (1.97-2.56)
Psychotic disorder	45 (0.1208)/139 (0.07655)	1.57 (1.06-2.34)	18 (0.1348)/55 (0.08428)	1.66 (0.83-3.33)	27 (0.1126)/85 (0.07287)	1.37 (0.81-2.33)

^a^Per 1000 person years. ^b^Based on the matched cohort study, HR (95% CI) was derived from the Cox regression models, stratified by matching identifier (birth year, sex), and adjusted for ethnicity, educational attainment, smoking status, drinking status, annual household income, Townsend's deprivation index (as a continuous variable), body mass index, physical activity, history of other psychiatry disorders, and Charlson's comorbidity index. Abbreviation: HR: hazard ratios; CI: confidence interval; OA: osteoarthritis.

**Table 2:** The genetic association between psychiatric disorders and osteoarthritis (OA) assessed by polygenic risk score (PRS) analysis.

Specific psychiatric disorders	Hip/knee OA				Hip OA				Knee OA			
	Psychiatric disorders PRS→OA		OA PRS→psychiatric disorders		Psychiatric disorders PRS→OA		OA PRS→psychiatric disorders		Psychiatric disorders PRS→OA		OA PRS→psychiatric disorders	
	OR (95% CI)^a^	*p*	OR (95% CI)^a^	*p*	OR (95% CI)^a^	*p*	OR (95% CI)^a^	*p*	OR (95% CI)^a^	*p*	OR (95% CI)^a^	*p*
Depression	1.03 (1.01-1.04)	3.31⁣^∗^10^−06^	1.03 (1.02-1.05)	1.22⁣^∗^10^−04^	1.00 (0.99-1.02)	0.642	1.01 (1.00-1.03)	0.068	1.04 (1.03-1.05)	8.52⁣^∗^10^−09^	1.04 (1.02-1.05)	1.97⁣^∗^10^−05^
Anxiety	1.01 (1.00-1.02)	0.0458	1.07 (1.05-1.09)	5.65⁣^∗^10^−14^	1.01 (0.99-1.02)	0.448	1.03 (1.01-1.05)	2.39⁣^∗^10^−04^	1.01 (1.00-1.02)	0.173	1.06 (1.04-1.08)	4.43⁣^∗^10^−12^
Stress-related disorder	1.03 (1.02-1.05)	5.95⁣^∗^10^−10^	1.03 (1.01-1.06)	0.0111	1.01 (1.00-1.03)	0.161	1.01 (0.98-1.03)	0.593	1.04 (1.03-1.06)	2.26⁣^∗^10^−11^	1.04 (1.01-1.06)	2.23⁣^∗^10^−03^
Substance misuse	1.00 (0.99-1.01)	0.853	1.07 (1.05-1.09)	1.24⁣^∗^10^−14^	0.99 (0.98-1.01)	0.439	1.02 (1.00-1.04)	0.013	1.01 (0.99-1.02)	0.348	1.08 (1.06-1.09)	2.23⁣^∗^10^−15^

^a^ORs and 95% CIs (per standard deviation increase in the corresponding PRS) were estimated by logistic regression models adjusting for age, sex, genotyping array, and the first ten ancestry principal components. Abbreviation: OA: osteoarthritis; OR: odds ratio; CI: confidence interval; PRS: polygenic risk score.

**Table 3:** Genetic association between depression and osteoarthritis (OA) assessed by LD score regression.

	Hip/knee OA		Hip OA		Knee OA	
	rg (95% CI)^a^	*p*	rg (95% CI)^a^	*p*	rg (95% CI)^a^	*p*
Depression	0.17 (0.12~0.22)	1.33⁣^∗^10^−11^	0.06 (0.00~0.12)	0.0488	0.20 (0.15~0.25)	1.75⁣^∗^10^−15^
Anxiety	0.07 (0.10~0.25)	0.4178	0.06 (-0.14~0.25)	0.5638	0.06 (-0.12~0.24)	0.5196
Stress-related disorder	0.28 (0.17~0.39)	3.76⁣^∗^10^−07^	0.15 (0.02~0.29)	0.0295	0.29 (0.19~0.40)	9.12⁣^∗^10^−08^
Substance misuse	-0.02 (-0.14~0.10)	0.7629	0.02 (-0.10~0.14)	0.7470	-0.07 (-0.20~0.07)	0.3220

^a^Genetic association was assessed by LD score regression. rg: *r* for genetic; CI: confidence interval.

## Data Availability

Data from the UK Biobank (http://www.ukbiobank.ac.uk/) are available to all researchers upon making an application. Part of this research was conducted using the UK Biobank Resource under Application 54803.
