# Supplementary material for: Genetic Determinants of the Association between Osteoarthritis and Psychiatric Disorders
Source: Depress Anxiety. 2023 Aug 3;2023:5253920. doi: 10.1155/2023/5253920 (PMC11921858; doi:10.1155/2023/5253920)
Supplement: Supplementary 1 — Supplementary methods. [file 5253920.f1.docx]

**Supplementary methods**

**Data source**

The UK Biobank is a large community-based cohort enrolled more than 0.5 million individuals aged 40 to 69 years across England, Scotland, and Wale between 2006 and 2010^1^. Participants filled out questionnaires about their sociodemographic characteristics, lifestyle, medical history, and other health-related factors at recruitment. In addition, blood samples were collected at baseline and the genotyping were carried out using Applied Biosystems UK BiLEVE Axiom Array and UK Biobank Axiom Array^1^. The imputed genotype dataset contained more than 97 million single nucleotide polymorphisms (SNPs) for 488,377 individuals. The UK Biobank also provided the kinship coefficient and principal components (PCs) for genetic analysis. Details regarding the genotyping and quality-control procedures for the UK Biobank have been previously published^1^. Follow-up data were obtained through periodically linked data from multiple national datasets, including death registries, primary and inpatient hospital records. In brief, the inpatient hospital data covered all UK Biobank participants in January 1997 and onwards, while the primary care data were obtained from multiple general practice data system suppliers, covering approximately 45% of the UK biobank participants^1^.

We identified the recent publicly available GWAS summary statistics results for OA and the five specific psychiatric disorders with the largest samples size of European ancestry^2-7^ in our genetic analyses. For OA and depression, of which the GWASs with largest sample included UK Biobank participants, to avoid sample overlap between the discovery and target data during PRSs calculation, we used GWAS for OA conducted by arcOGEN Consortium^8^, and the GWAS summary data for depression that removed UK Biobank participants^9^, as the independent data resource for SNP selection and risk allele weighting. Details regarding the numbers of individuals and SNPs included in these GWASs are summarized in the Supplementary Table 1.

**Phenotypic association analyses in UK Biobank**

All Cox models stratified by matched factors (birth year and sex) and adjusted for ethnicity (White, others or unknown), body mass index (BMI, <18.5, 18.5-24.9, 25-29.9, ≥30.0, or unknown), smoking and drinking status (never, previous, current, or unknown), annual household income (<£18,000, £18,000-30,999, £31,000-51,999, £52,000-100,000, >£100,000, or unknown), educational attainment (college degree, A-level, O-level, Certificate of Secondary Education or equivalent, National Vocation Qualifications or equivalent, other professional qualifications, or unknown), Townsend deprivation index (as a continuous variable), physical activity (low, moderate, high, or unknown), history of other psychiatry disorders (yes, or no), and Charlson comorbidity index (0, 1, ≥2)^10^.

**Quality control before P*RS analyses***

To generate the PRSs for OA and subtypes of psychiatry disorder, we included autosomal biallelic SNPs and removed variants with a call rate < 98%, minor allele frequency < 0.01, or deviation from Hardy–Weinberg equilibrium (p < 10^−6^). We also removed individuals having a genotyping rate < 98%, related individuals (up to the third degree, i.e., kinship coefficient > 0.044), and outlier samples based on their abnormal heterozygosity level. After this process, 338,573 participants with 7,130,905 SNPs were retained for further analysis. The details of the quality-control strategy are summarized in Supplementary Figure 3.

For the GWAS summary data, we further restricted to SNPs available in both base data set (i.e., GWAS summary data) and target data set (i.e., UK Biobank genotypic data that passed mentioned quality control), and excluded the variants with ambiguous strand and LD threshold of r^2^ > 0.1 in 250kb window. Then, we computed PRSs as the sum of SNP dosages weighted by the allele effect across all SNPs in the target data set under a GWAS-*p* threshold of 5 × 10^−8^, 1 × 10^−6^, 1 × 10^−4^, 1 × 10^−3^, 0.01, 0.05, 0.1, 0.2, 0.3, 0.4, and 0.5, respectively. PLINK (version 1.9) was used for the PRS profiling.

**Reference**

1. Bycroft C, Freeman C, Petkova D, Band G, Elliott LT, Sharp K, et al. The UK Biobank resource with deep phenotyping and genomic data. *Nature* 2018; **562**(7726): 203-9.

2. Tachmazidou I, Hatzikotoulas K, Southam L, Esparza-Gordillo J, Haberland V, Zheng J, et al. Identification of new therapeutic targets for osteoarthritis through genome-wide analyses of UK Biobank data. *Nat Genet* 2019; **51**(2): 230-6.

3. Stringer S, Minica CC, Verweij KJ, Mbarek H, Bernard M, Derringer J, et al. Genome-wide association study of lifetime cannabis use based on a large meta-analytic sample of 32 330 subjects from the International Cannabis Consortium. *Transl Psychiatry* 2016; **6**: e769.

4. Schizophrenia Working Group of the Psychiatric Genomics C. Biological insights from 108 schizophrenia-associated genetic loci. *Nature* 2014; **511**(7510): 421-7.

5. Otowa T, Hek K, Lee M, Byrne EM, Mirza SS, Nivard MG, et al. Meta-analysis of genome-wide association studies of anxiety disorders. *Molecular psychiatry* 2016; **21**(10): 1391-9.

6. Meier SM, Trontti K, Purves KL, Als TD, Grove J, Laine M, et al. Genetic Variants Associated With Anxiety and Stress-Related Disorders: A Genome-Wide Association Study and Mouse-Model Study. *JAMA psychiatry* 2019; **76**(9): 924-32.

7. Howard DM, Adams MJ, Clarke TK, Hafferty JD, Gibson J, Shirali M, et al. Genome-wide meta-analysis of depression identifies 102 independent variants and highlights the importance of the prefrontal brain regions. *Nat Neurosci* 2019; **22**(3): 343-52.

8. arc OC, arc OC, Zeggini E, Panoutsopoulou K, Southam L, Rayner NW, et al. Identification of new susceptibility loci for osteoarthritis (arcOGEN): a genome-wide association study. *Lancet* 2012; **380**(9844): 815-23.

9. Wray NR, Ripke S, Mattheisen M, Trzaskowski M, Byrne EM, Abdellaoui A, et al. Genome-wide association analyses identify 44 risk variants and refine the genetic architecture of major depression. *Nat Genet* 2018; **50**(5): 668-81.

10. Quan H, Sundararajan V, Halfon P, Fong A, Burnand B, Luthi JC, et al. Coding algorithms for defining comorbidities in ICD-9-CM and ICD-10 administrative data. *Med Care* 2005; **43**(11): 1130-9.
