# Supplementary material for: Genetic Determinants of the Association between Osteoarthritis and Psychiatric Disorders
Source: Depress Anxiety. 2023 Aug 3;2023:5253920. doi: 10.1155/2023/5253920 (PMC11921858; doi:10.1155/2023/5253920)
Supplement: Supplementary 2 — Supplementary tables and figures. [file 5253920.f2.docx]

Supplementary Table 1 Genome-wide Association Study (GWAS) summary statistics in this study

Supplementary Table 2 International Classification of Diseases (ICD) and primary care data READ v2/v3 codes for diagnoses used in this study

Supplementary Table 3 The association between polygenic risk scores for osteoarthritis under different p value thresholds and the corresponding trait

Supplementary Table 4 The association between polygenic risk scores for osteoarthritis/depression under different p value thresholds and the corresponding trait

Supplementary Table 5 The association between psychiatric disorders and subsequent risk of osteoarthritis (OA) by source of exposure identification

Supplementary Table 6 The association between osteoarthritis (OA) and subsequent risk of psychiatric disorders by source of exposure identification

Supplementary Table 7 Shared risk genes between knee osteoarthritis and depression/stress-related disorder

Supplementary Figure 1 Study flowchart for the matched cohort assessing the association of psychiatry disorders with subsequent osteoarthritis

Supplementary Figure 2 Study flowchart for the matched cohort assessing the association of osteoarthritis with subsequent psychiatry disorders

Supplementary Figure 3 Study flowchart for PRS analyses

**Supplementary Table 1 Genome-wide Association Study (GWAS) summary statistics in this study**

| **Trait** | **Sample size** | **SNP (millions)** | **First author, year (PMID)** |
| --- | --- | --- | --- |
| Publicly available GWAS data with largest sample sizes | | | |
| Depression | 500,199 | 8.5 | Howard D, 2019 (30718901) |
| Anxiety | 17,310 | 6.5 | Otowa, T, 2016 (26754954) |
| Stress-related disorder | 29,056 | 8.9 | Meier, S, 2019 (31116379) |
| Substance misuse | 32,330 | 6.4 | Stringer, S, 2016 (27023175) |
| Psychotic disorder | 77,096 | 11.3 | Schizophrenia Working Group of the PGC, 2014 (25056061) |
| Hip/knee osteoarthritis | 417,596 | 17.5 | Tachmazidou I, 2019 (30664745) |
| Hip osteoarthritis | 393,873 |  |  |
| Knee osteoarthritis | 403,124 |  |  |
| Independent sample of UK Biobank, used for PRS calculation | | | |
| Depression | 142,646 | 9.9 | Wray N, 2018 (29700475) |
| Hip/knee osteoarthritis | 18,419 | 11.3 | arcOGEN, 2012 (22763110) |
| Hip osteoarthritis | 14,275 |  |  |
| Knee osteoarthritis | 14,507 |  |  |

SNP: single nucleotide polymorphisms; GWAS: genome-wide association studies

**Supplementary Table 2 International Classification of Diseases (ICD) and primary care data READ v2/v3 codes for diagnoses used in this study**

| **Disease** | **ICD-10** | **ICD-9** | **Read V2** | **Read V3** |
| --- | --- | --- | --- | --- |
| Depression | F32-F33 | 2961, 3004, 311 | E112., E1120, E1121, E1122, E1123, E1124, E1125, E1126, E112z, E113., E1130, E1131, E1132, E1133, E1134, E1135, E1136, E1137, E113z, E135., Eu32., Eu320, Eu321, Eu322, Eu323, Eu324, Eu325, Eu326, Eu327, Eu328, Eu329, Eu32A, Eu32B, Eu32y, Eu32z, Eu33., Eu330, Eu331, Eu332, Eu333, Eu334, Eu33y, Eu33z | 2257, E0043, E1120, E1121, E1122, E1123, E1124, E1125, E1126, E112z, E1130, E1131, E1132, E1133, E1134, E1135, E1136, E1137, E113z, E11y2, E130, E2B0., E2B1., Eu320, Eu321, Eu322, Eu323, Eu32y, Eu32z, Eu330, Eu331, Eu332, Eu333, Eu334, Eu33y, Eu33z, X00SO, X00SQ, X00SS, X00SU, Xa0wV, XaB9J, XaCHo, XaCHr, XaCHs, XaCIs, XaCIt, XaCIu, XaX53, XaX54, XaY2C, XE1Y0, XE1Y1, XE1YC, XE1Za, XE1Zb, XE1Zc, XE1Zd, XE1Ze, XE1Zf, XE1ZY, XE1ZZ, XM1GC, XSEGJ, XSGok, XSGol, XSGom, XSGon |
| Anxiety | F40, F41 | 3000, 3002 | E2000, E2001, E2002, E2004, E2005, E200z, E202., E2020, E2021, E2022, E2023, E2024, E2025, E2026, E2027, E2028, E2029, E202A, E202B, E202C, E202D, E202E, E202z, Eu40., Eu400, Eu401, Eu402, Eu403, Eu40y, Eu40z, Eu41., Eu410, Eu411, Eu412, Eu413, Eu41y, Eu41z | E200., E2000, E2002, E2004, E200z, E2020, E2021, E2022, E2023, E2024, E2025, E2026, E2027, E2028, E2029, E202E, Eu40., Eu400, Eu402, Eu40y, Eu40z, Eu41., Eu410, Eu413, Eu41y, Eu41z, Eu931, Ua1qa, Ua1qc, Ua1qd, Ua1qe, Ua1qf, Ua1qg, Ua1qh, Ua1qi, Ua1qj, Ua1qk, Ua1ql, , Ua1qm, Ua1qn, Ua1qo, Ua1qp, Ua1qs, Ua1qt, Ua1qU, Ua1qV, Ua1qW, Ua1qX, Ua1qY, X00RP, X00Sa, X00Sb, X00Sc, X00Sd, X00Se, X00Sr, X00SV, X00SW, X00SX, X00SY, X00SZ, X50G2, X50G3, X50G5, X50G6, X50GI, X75YV, X761d, X761n, X761q, X761t, X761u, X761y, X7627, X7628, X7629, X762a, X762C, X762E, X762F, X762G, X762H, X762T, X762Z, X78wp, Xa00r, Xa00s, Xa1a8, Xa1Ev, Xa3Vj, Xa3Vk, Xa3Vl, Xa3WH, Xa3WI, Xa3WJ, Xa7k9, Xa7kB, XaIo7, XaKVA, XE0rb, XE1Y7, XE1YA, XE1YB, XE1Zj, XM0Ak |
| Stress-related disorder | F43 | 308, 309 | E280., E281., E282., E283., E2830, E2831, E283z, E284., E28z., E2900, E2925, E292y, E292z, E293., E2930, E2931, E2932, E293z, E294., E29y., E29y1, E29y2, E29y4, E29yz, E29z., Eu43., Eu430, Eu431, Eu432, Eu433, Eu434, Eu435, Eu43y, Eu43z | 1B1L., 1BE.., E280., E281., E282., E283., E2830, E2831, E283z, E284., E29.., E290., E290z, E291., E292., E2920, E2921, E2922, E2924, E2925, E292y, E292z, E293., E2930, E2931, E2932, E293z, E294., E29y., E29y0, E29y1, E29y2, E29y3, E29y4, E29y5, E29yz, E29z., Eu430, Eu432, Eu43y, Eu43z, Eu930, Ry15., Ua18k, Ua18L, Ub1T9, X00Sf, X00TT, X40Js, Xa028, Xa18j, Xa18v, XaC2u, XaX55, XaX56, XaX58, XE1Ym, XE1Yn, XE1Yo, XE1Yp, XE2uz, XM0As, XM1Q3 |
| Substance misuse | F10-F19 | 291, 303-305 | E01.., E010., E011., E0110, E0111, E0112, E011z, E012., E0120, E013., E014., E015., E01y., E01y0, E01yz, E01z., E23.., E230., E2300, E2301, E2302, E2303, E230z, E231., E2310, E2311, E2312, E2313, E231z, E23z., E24.., E240., E2400, E2401, E2402, E2403, E240z, E241., E2410, E2411, E2412, E2413, E241z, E242., E2420, E2421, E2422, E2423, E242z, E243., E2430, E2431, E2432, E2433, E243z, E244., E2440, E2441, E2442, E2443, E244z, E245., E2450, E2451, E2452, E2453, E245z, E246., E2460, E2461, E2462, E2463, E246z, E247., E2470, E2471, E2472, E2473, E247z, E248., E2480, E2481, E2482, E2483, E248z, E249., E2490, E2491, E2492, E2493, E249z, E24A., E24z., E25.., E250., E2500, E2501, E2502, E2503, E250z, E251., E2510, E2511, E2512, E2513, E251z, E252., E2520, E2521, E2522, E2523, E252z, E253., E2530, E2531, E2532, E2533, E253z, E254., E2540, E2541, E2542, E2543, E254z, E255., E2550, E2551, E2552, E2553, E255z, E256., E2560, E2561, E2562, E2563, E256z, E257., E2570, E2571, E2572, E2573, E257z, E258., E2580, E2581, E2582, E2583, E258z, E259., E2590, E2591, E2592, E2593, E2594, E259z, E25y., E25y0, E25y1, E25y2, E25y3, E25yz, E25z., Eu1.., Eu10., Eu100, Eu101, Eu102, Eu103, Eu104, Eu105, Eu106, Eu107, Eu108, Eu10y, Eu10z, Eu11., Eu110, Eu111, Eu112, Eu113, Eu114, Eu115, Eu116, Eu117, Eu11y, Eu11z, Eu12., Eu120, Eu121, Eu122, Eu123, Eu124, Eu125, Eu126, Eu127, Eu12y, Eu12z, Eu13., Eu130, Eu131, Eu132, Eu133, Eu134, Eu135, Eu136, Eu137, Eu13y, Eu13z, Eu14., Eu140, Eu141, Eu142, Eu143, Eu144, Eu145, Eu146, Eu147, Eu14y, Eu14z, Eu15., Eu150, Eu151, Eu152, Eu153, Eu154, Eu155, Eu156, Eu157, Eu15y, Eu15z, Eu16., Eu160, Eu161, Eu162, Eu163, Eu164, Eu165, Eu166, Eu167, Eu16y, Eu16z, Eu17., Eu170, Eu171, Eu172, Eu173, Eu174, Eu175, Eu176, Eu177, Eu17y, Eu17z, Eu18., Eu180, Eu181, Eu182, Eu183, Eu184, Eu185, Eu186, Eu187, Eu18y, Eu18z, Eu19., Eu190, Eu191, Eu192, Eu193, Eu194, Eu195, Eu196, Eu197, Eu19y, Eu19z, Eu1A., Eu1A0, Eu1A1, Eu1A2, Eu1A3, Eu1A4, Eu1A5, Eu1A6, Eu1A7, Eu1Ay, Eu1Az, | 136K., 137H., 137J., 137R., E01.., E010., E011., E0111, E011z, E0120, E013., E014., E015., E01y0, E02.., E021., E0210, E0211, E023., E02y4, E230., E2301, E2302, E230z, E2311-E2313, E24.., E2401-E2403, E2411-E2413, E242., E2421-E2423, E2431-E2433, E2441-E2443, E2450-E2453, E246., E2460-E2463, E248., E2481-E2483, E249., E2491-E2493, E24A., E2501-E2503, E251., E2511-E2513, E252., E2521-E2523, E2531,E2532, E2541, E2542, E255., E2551-E2553, E256., E2561-E2563, E2571,E2572, E259., E2591-E2593, Eu10., Eu100-Eu107, Eu10y, Eu10z, Eu11., Eu110-Eu117, Eu11y, Eu11z, Eu12., Eu13., Eu14., Eu15., Eu152, Eu16., Eu167, Eu17., Eu18., Eu19., Eu192, Ub00U, Ub0ly, Ub0lz, Ub0ma, Ub0mt-Ub0mw, Ub0mX, Ub0mY, Ub1tS-Ub1tW, X00Rg-X00Rz, X00S0-X00S2, X401f, X711m, X75yW, X767V, Xa17e, Xa71m, Xa1yZ, Xa25J, Xa2lt, Xa2m3, XaA1V, XabbR, Xabi7-Xabi9, XaBZA, XaevR, XaKBz, XaKC0, XaKC1, XaKC4, XaKC8, XaKUQ-XaKUS, XaKV9, XakvA, XaKvB, XaLQK, XaLQN, XaLTr, XaLWu, XaMhO, XaPQC, XE1Ya, XE1YQ-XE1YT, XE1YV, XE1YX-XE1YZ, XE1ZE-XE1ZI, XM0qQ, XM1Q0 |
| Psychotic disorder | F20-F29 | 295, 297, 298 | E042., E1..., E10.., E100., E1000, E1001, E1002, E1003, E1004, E1005, E100z, E101., E1010, E1011, E1012, E1013, E1014, E1015, E101z, E102., E1020, E1021, E1022, E1023, E1024, E1025, E102z, E103., E1030, E1031, E1032, E1033, E1034, E1035, E103z, E104., E105., E1050, E1051, E1052, E1053, E1054, E1055, E105z, E106., E107., E1070, E1071, E1072, E1073, E1074, E1075, E107z, E10y., E10y0, E10y1, E10yz, E10z., E12.., E120., E121., E122., E123., E12y., E12y0, E12yz, E12z., E13.., E130., E131., E132., E133., E134., E13y., E13y0, E13y1, E13yz, E13z., E1y.., E1z.., R009., R00zX, Eu2.., Eu20., Eu200, Eu201, Eu202, Eu203, Eu204, Eu205, Eu206, Eu20y, Eu20z, Eu21., Eu22., Eu220, Eu221, Eu222, Eu223, Eu22y, Eu22z, Eu23., Eu230, Eu231, Eu232, Eu233, Eu23y, Eu23z, Eu24., Eu25., Eu250, Eu251, Eu252, Eu25y, Eu25z, Eu26., Eu2y., Eu2z. | 1BC.., E03y0, E1…, E10.., E100, E1001, E1003-E1005, E101, E1011-E1015, E102., E1021-E1025, E103., E1031-E1035, E105., E1051-E1055, E106., E107., E1071-E1075, E10y0, E10y1, E120., E122., E123., E124., E13y1, E2122, Eu20., Eu202, Eu203, Eu20y, Eu20z, Eu21., Eu220, Eu22y, Eu22z, Eu230-Eu233, Eu23y, Eu23z, Eu24., Eu25., Eu25y, Eu25z, Eu2y., Ua1rH, Ua1WW, X00Qy, X00RL, X00RV, X00S8, X00SA, X00SC, X00SD, X50GE-X50GH, X50GJ-X50GL, X75yp, X75z7, X761M, X763T, Xa00v, Xa0s9, Xa0tC, Xa33a, Xa33b, XaB8j, XaX52, XE1Xw, XE1Y2, XE1Y4, XE1ZM-XE1ZU, XE2b8, XE2un, XE2uT, XM1GG, XM1GH, XSKr7 |
| Hip osteoarthritis | M16 | 71515, 71525, 71515, 71595 | N0515, N0519, N051A, N0529, N0545, N05z5 N05zJ, Nyu21, Nyu22, Nyu23, Nyu24, Nyu2E, N0515, N0519, N051A, N0525, N0529, N0535, N05z5, N05zJ | N0515, N0519, N051A, N0529, N0545, N05z5, N05zJ, Nyu21, Nyu22, Nyu23, Nyu24, Nyu2E, XE1De, N0515, N0519, N051A, N0529, N05zJ, X7007, XE1De |

**Supplementary Table 3 The association between polygenic risk scores for osteoarthritis under different p value thresholds and the corresponding trait**

| Pt | Hip/knee osteoarthritis | | | | Hip osteoarthritis | | | | Knee osteoarthritis | | | |
| --- | --- | --- | --- | --- | --- | --- | --- | --- | --- | --- | --- | --- |
|  | N_SNP | OR (95% CI) ^a^ | R^2^ | p value | N_SNP | OR (95% CI) ^a^ | R^2^ | p value | N_SNP | OR (95% CI) | R^2^ | p value |
| Pt5e^-08^ | 2 | 1.02 (1.01-1.03) | 4.783 | 4.23×10^-4^ | 2 | 1.04 (1.03-1.06) | 4.517 | 1.52×10^-7^ | / | / | / | / |
| Pt1e^-06^ | 2 | 1.02 (1.01-1.03) | 4.783 | 4.23×10^-4^ | 6 | 1.06 (1.05-1.08) | 4.539 | 4.23×10^-14^ | 8 | 1.01 (1.00-1.02) | 3.792 | 0.186 |
| Pt1e^-04^ | 194 | 1.04 (1.03-1.05) | 4.798 | 5.1×10^-12^ | 204 | 1.10 (1.08-1.12) | 4.599 | 6.87×10^-32^ | 148 | 1.03 (1.01-1.04) | 3.799 | 1.32×10^-4^ |
| Pt1e^-03^ | 1356 | 1.07 (1.06-1.08) | 4.842 | 6.45×10^-36^ | 1128 | 1.10 (1.08-1.11) | 4.594 | 4.02×10^-30^ | 1164 | 1.07 (1.05-1.08) | 3.845 | 3.84×10^-23^ |
| Pt0.05 | 28758 | 1.13 (1.11-1.14) | 4.939 | 2.58×10^-87^ | 26628 | 1.16 (1.14-1.18) | 4.742 | 8.87×10^-74^ | 27866 | 1.14 (1.12-1.15) | 3.967 | 6.98×10^-72^ |
| Pt0.1 | 47764 | 1.13 (1.12-1.15) | 4.952 | 4.44×10^-94^ | 45524 | 1.18 (1.16-1.20) | 4.785 | 2.42×10^-86^ | 46896 | 1.15 (1.14-1.17) | 3.994 | 1.61×10^-82^ |
| Pt0.2 | 78664 | 1.15 (1.13-1.16) | 4.970 | 7.35×10^-104^ | 76162 | 1.18 (1.16-1.20) | 4.795 | 1.72×10^-89^ | 77690 | 1.16 (1.14-1.18) | 4.009 | 1.14×10^-88^ |
| Pt0.3 | **103488** | **1.15 (1.13-1.16)** | **4.975** | 9.41×10^-107^ | 101482 | 1.19 (1.17-1.21) | 4.807 | 5.58×10^-93^ | 103066 | 1.17 (1.15-1.18) | 4.019 | 2.74×10^-92^ |
| Pt0.4 | 124644 | 1.15 (1.14-1.16) | 4.974 | 3.13×10^-106^ | **122882** | **1.19 (1.17-1.21)** | **4.818** | 2.55×10^-96^ | **124542** | **1.17 (1.15-1.19)** | **4.023** | 5.79×10^-94^ |
| Pt0.5 | 143020 | 1.15 (1.13-1.16) | 4.972 | 1.03×10^-104^ | 141332 | 1.19 (1.17-1.21) | 4.814 | 4.26×10^-95^ | 142896 | 1.17 (1.15-1.19) | 4.022 | 1.05×10^-93^ |

OR were adjusting for age, sex, genotyping array, and the first 10 ancestry principal components. OR: odds ratio; CI: confidence interval

Pt was p value threshold. N_SNP was the number of SNP used predictive model. R2 was Nagelkerke’s squared (R square).

**Supplementary Table 4 The association between polygenic risk scores for osteoarthritis/depression under different p value thresholds and the corresponding trait**

| Pt | Depression | | | | Anxiety | | | | Stress-related disorder | | | | Substance misuse | | | |
| --- | --- | --- | --- | --- | --- | --- | --- | --- | --- | --- | --- | --- | --- | --- | --- | --- |
|  | N_SNP | OR (95% CI) ^a^ | R^2^ | p value | N_SNP | OR (95% CI) ^a^ | R^2^ | p value | N_SNP | OR (95% CI) | R^2^ | p value | N_SNP | OR (95% CI) ^a^ | R^2^ | p value |
| Pt5e^-08^ | 4 | 1.02 (1.01-1.04) | 1.180 | 3.17×10^-3^ | 2 | 1.00 (0.99-1.01) | 1.245 | 0.997 | 2 | 0.99 (0.97-1.01) | 1.930 | 0.445 | - | - | - | - |
| Pt1e^-06^ | 40 | 1.03 (1.01-1.04) | 1.183 | 2.65×10^-4^ | 2 | 1.00 (0.99-1.01) | 1.245 | 0.997 | 10 | 1.00 (0.98-1.03) | 1.929 | 0.667 | 2 | 1.00 (0.99-1.02) | 2.111 | 0.633 |
| Pt1e^-04^ | 642 | 1.05 (1.03-1.06) | 1.199 | 6.78×10^-10^ | 232 | 0.99 (0.98-1.01) | 1.246 | 0.206 | 388 | 1.03 (1.01-1.05) | 1.939 | 3.05×10^-3^ | 258 | 1.01 (0.99-1.02) | 2.112 | 0.336 |
| Pt1e^-03^ | 3282 | 1.08 (1.07-1.10) | 1.252 | 1.44×10^-27^ | 1570 | 1.02 (1.00-1.03) | 1.249 | 0.012 | 2334 | 1.05 (1.02-1.07) | 1.950 | 2.2×10^-5^ | 1904 | 1.01 (0.99-1.02) | 2.112 | 0.363 |
| Pt0.05 | 63658 | 1.13 (1.11-1.14) | 1.344 | 5.07×10^-58^ | 40714 | 1.04 (1.02-1.05) | 1.262 | 7.38×10^-7^ | 48954 | 1.09 (1.07-1.11) | 2.007 | 3.27×10^-16^ | 47448 | 1.04 (1.02-1.05) | 2.126 | 7.64×10^-6^ |
| Pt0.1 | 105786 | 1.13 (1.11-1.15) | 1.358 | 1.12×10^-62^ | **68142** | **1.04 (1.03-1.06)** | **1.267** | **1.03×10^-8^** | 81214 | 1.09 (1.07-1.11) | 2.006 | 5.28×10^-16^ | **79634** | **1.04 (1.02-1.06)** | **2.130** | **5.51×10^-7^** |
| Pt0.2 | **174028** | **1.14 (1.12-1.15)** | **1.370** | **8.93×10^-67^** | 108834 | 1.04 (1.02-1.05) | 1.262 | 5.41×10^-7^ | **133130** | **1.09 (1.07-1.11)** | **2.009** | **1.33×10^-16^** | 128186 | 1.04 (1.02-1.06) | 2.129 | 7.89×10^-7^ |
| Pt0.3 | 229256 | 1.13 (1.12-1.15) | 1.366 | 2.88×10^-65^ | 139632 | 1.04 (1.03-1.06) | 1.264 | 9.56×10^-8^ | 175438 | 1.09 (1.07-1.11) | 2.007 | 2.82×10^-16^ | 165478 | 1.04 (1.02-1.06) | 2.129 | 9.39×10^-7^ |
| Pt0.4 | 275196 | 1.13 (1.12-1.15) | 1.362 | 7.18×10^-64^ | 163478 | 1.04 (1.03-1.06) | 1.264 | 1.09×10^-7^ | 211658 | 1.09 (1.07-1.11) | 2.009 | 1.6×10^-16^ | 195356 | 1.04 (1.03-1.06) | 2.130 | 4.92×10^-7^ |
| Pt0.5 | 314454 | 1.13 (1.12-1.15) | 1.363 | 2.15×10^-64^ | 183200 | 1.04 (1.03-1.06) | 1.265 | 7.31×10^-8^ | 241866 | 1.09 (1.07-1.11) | 2.008 | 1.81×10^-16^ | 219554 | 1.04 (1.02-1.06) | 2.128 | 2.31×10^-6^ |

OR were adjusting for age, sex, genotyping array, and the first 10 ancestry principal components. OR: odds ratio; CI: confidence interval

Pt was p value threshold. N_SNP was the number of SNP used predictive model. R2 was Nagelkerke’s squared (R square).

**Supplementary Table 5 The association between psychiatric disorders and** **subsequent risk of osteoarthritis (OA) by source of exposure identification**

| Source of exposure identification | Hip/knee OA | | Hip OA | | Knee OA | |
| --- | --- | --- | --- | --- | --- | --- |
|  | No. of cases (incidence ^a^) in patients/matched individuals | HR (95% CI) ^b^ | No. of cases (incidence ^a^) in patients/matched individuals | HR (95% CI) ^b^ | No. of cases (incidence ^a^) in patients/matched individuals | HR (95% CI) ^b^ |
| Any psychiatric disorders |  |  |  |  |  |  |
| Only primary | 4994(8.763)/14992(5.317) | 1.65 (1.59-1.71) | 1680(2.821)/6552(2.279) | 1.22 (1.15-1.29) | 3637(6.282)/9265(3.247) | 1.92 (1.84-2.01) |
| Inpatient | 348(6.482)/1343(4.905) | 1.32 (1.15-1.52) | 136(2.466)/573(2.056) | 1.23 (0.99-1.54) | 228(4.187)/846(3.059) | 1.35 (1.13-1.61) |
| Depression |  |  |  |  |  |  |
| Only primary | 1574(9.191)/4237(5.004) | 1.71 (1.60-1.82) | 524(2.92)/1889(2.19) | 1.29 (1.16-1.43) | 1149(6.594)/2592(3.026) | 1.95 (1.81-2.11) |
| Inpatient | 113(6.623)/417(4.906) | 1.28 (1.00-1.65) | 44(2.507)/175(2.019) | 1.08 (0.71-1.64) | 71(4.089)/269(3.131) | 1.41 (1.02-1.95) |
| Anxiety |  |  |  |  |  |  |
| Only primary | 1342(8.43)/4186(5.363) | 1.61 (1.51-1.72) | 435(2.614)/1851(2.326) | 1.14 (1.02-1.27) | 1003(6.212)/2571(3.254) | 1.93 (1.78-2.09) |
| Inpatient | 64(9.616)/228(6.727) | 1.34 (0.97-1.84) | 25(3.637)/93(2.69) | 1.40 (0.83-2.36) | 42(6.218)/146(4.262) | 1.36 (0.90-2.05) |
| Stress-related disorder |  |  |  |  |  |  |
| Only primary | 766(8.845)/2224(5.199) | 1.65 (1.51-1.80) | 251(2.77)/974(2.234) | 1.20 (1.04-1.39) | 561(6.372)/1370(3.166) | 1.96 (1.76-2.19) |
| Inpatient | 23(6.539)/70(4.01) | 1.38 (0.72-2.68) | 5(1.375)/31(1.751) | 0.99 (0.28-3.53) | 19(5.359)/44(2.496) | 1.90 (0.79-4.58) |
| Substance misuse |  |  |  |  |  |  |
| Only primary | 1328(8.566)/4391(5.67) | 1.66 (1.53-1.79) | 471(2.919)/1859(2.351) | 1.30 (1.15-1.49) | 939(5.965)/2761(3.522) | 1.89 (1.71-2.09) |
| Inpatient | 79(5.906)/332(4.601) | 1.33 (0.96-1.85) | 37(2.699)/144(1.964) | 1.77 (1.07-2.93) | 47(3.46)/204(2.801) | 1.17 (0.76-1.81) |
| Psychotic disorder |  |  |  |  |  |  |
| Only primary | 15(6.04)/60(4.759) | 1.56 (0.70-3.49) | 2(0.7736)/28(2.185) | 0.13 (0.02-1.15) | 13(5.214)/35(2.748) | 2.63 (0.85-8.12) |
| Inpatient | 17(2.335)/160(4.433) | 0.63 (0.34-1.14) | 8(1.091)/74(2.024) | 0.90 (0.35-2.33) | 11(1.505)/94(2.578) | 0.51 (0.22-1.17) |

a. Per 1000 person years.

b. Based on the matched cohort study, HR (95% CI) were derived from Cox regression models, stratified by matching identifier (birth year, sex), and adjusted for ethnicity, educational attainment, smoking status, drinking status, annual household income, Townsend deprivation index (as a continuous variable), Body Mass Index, physical activity, history of other psychiatry disorders, and Charlson comorbidity index.

Abbreviation: HR: Hazard ratios; CI: confidence interval; OA: osteoarthritis.

**Supplementary Table 6 The association between osteoarthritis (OA) and subsequent risk of psychiatric disorders** **by source of exposure identification**

| Source of exposure identification | Any psychiatric disorders | | Depression | | Anxiety | | Stress-related disorder | | Substance misuse | | Psychotic disorder | |
| --- | --- | --- | --- | --- | --- | --- | --- | --- | --- | --- | --- | --- |
|  | No. of cases (incidence ^a^) in patients/matched individuals | HR  (95% CI) ^b^ | No. of cases (incidence ^a^) in patients/matched individuals | HR  (95% CI) ^b^ | No. of cases (incidence ^a^) in patients/matched individuals | HR  (95% CI) ^b^ | No. of cases (incidence ^a^) in patients/matched individuals | HR  (95% CI) ^b^ | No. of cases (incidence ^a^) in patients/matched individuals | HR  (95% CI) ^b^ | No. of cases (incidence ^a^) in patients/matched individuals | HR  (95% CI) ^b^ |
| Hip/knee OA |  |  |  |  |  |  |  |  |  |  |  |  |
| Only primary | 874(11)/1330(3.27) | 3.49  (3.18-3.84) | 258(3.02)/395(0.928) | 2.98  (2.52-3.52) | 271(3.18)/358(0.841) | 3.79  (3.20-4.48) | 160(1.86)/185(0.435) | 4.13  (3.30-5.18) | 307(3.61)/455(1.07) | 3.84  (3.17-4.67) | 10(0.114)/27(0.0635) | 0.89  (0.24-3.24) |
| Inpatient | 1217(4.44)/3942(2.96) | 1.45  (1.35-1.55) | 444(1.58)/1123(0.808) | 1.76  (1.56-1.97) | 364(1.29)/1103(0.793) | 1.58  (1.40-1.79) | 180(0.635)/478(0.344) | 1.70  (1.41-2.04) | 412(1.46)/1460(1.05) | 1.32  (1.15-1.51) | 35(0.123)/112(0.0806) | 1.72  (1.09-2.71) |
| Hip OA |  |  |  |  |  |  |  |  |  |  |  |  |
| Only primary | 160(10.1)/269(3.36) | 3.27  (2.62-4.08) | 53(3.15)/78(0.928) | 3.64  (2.43-5.46) | 55(3.28)/67(0.797) | 4.23  (2.83-6.32) | 32(1.88)/39(0.464) | 4.11  (2.36-7.13) | 39(2.3)/104(1.24) | 2.01  (1.23-3.26) | 4(0.231)/4(0.0476) | 16441599.54 (0.00-Inf) |
| Inpatient | 380(3.4)/1558(2.9) | 1.13  (1.01-1.27) | 137(1.2)/424(0.754) | 1.52  (1.24-1.87) | 104(0.911)/464(0.826) | 1.10  (0.88-1.37) | 50(0.437)/189(0.336) | 1.23  (0.89-1.71) | 141(1.24)/580(1.03) | 1.09  (0.87-1.36) | 14(0.122)/51(0.0907) | 1.34  (0.63-2.88) |
| Knee OA |  |  |  |  |  |  |  |  |  |  |  |  |
| Only primary | 723(11.3)/1068(3.25) | 3.61  (3.25-4.01) | 209(3.03)/319(0.929) | 2.96  (2.46-3.57) | 217(3.15)/293(0.853) | 3.76  (3.11-4.55) | 130(1.87)/146(0.425) | 4.31  (3.33-5.58) | 270(3.94)/354(1.03) | 4.68  (3.75-5.84) | 6(0.0848)/24(0.0699) | 0.54  (0.12-2.35) |
| Inpatient | 777(4.92)/2311(3) | 1.61  (1.48-1.77) | 287(1.77)/675(0.84) | 1.83  (1.57-2.13) | 241(1.48)/623(0.775) | 1.86  (1.58-2.18) | 117(0.714)/278(0.346) | 1.96  (1.55-2.49) | 251(1.54)/854(1.06) | 1.47  (1.24-1.76) | 20(0.121)/58(0.0722) | 2.19  (1.10-4.36) |

a. Per 1000 person years.

b. Based on the matched cohort study, HR (95% CI) were derived from Cox regression models, stratified by matching identifier (birth year, sex), and adjusted for ethnicity, educational attainment, smoking status, drinking status, annual household income, Townsend deprivation index (as a continuous variable), Body Mass Index, physical activity, history of other psychiatry disorders, and Charlson comorbidity index.

Abbreviation: HR: Hazard ratios; CI: confidence interval; OA: osteoarthritis.

**Supplementary Table 7 Shared risk genes between knee osteoarthritis and depression/****stress-related disorder**

| ENSG | Symbol | CHR | Start | End | Strand | Type | EntrezID |
| --- | --- | --- | --- | --- | --- | --- | --- |
| Depression and knee OA | | | | | | | |
| ENSG00000026950 | BTN3A1 | 6 | 26402465 | 26415444 | 1 | protein_coding | 11119 |
| ENSG00000111801 | BTN3A3 | 6 | 26440700 | 26453643 | 1 | protein_coding | 10384 |
| ENSG00000112763 | BTN2A1 | 6 | 26458150 | 26476849 | 1 | protein_coding | 11120 |
| ENSG00000124549 | BTN2A3P | 6 | 26421619 | 26432611 | 1 | pseudogene | 54718 |
| ENSG00000124557 | BTN1A1 | 6 | 26501449 | 26510650 | 1 | protein_coding | 696 |
| ENSG00000124578 | HIST1H4G | 6 | 26246886 | 26247259 | -1 | protein_coding | 8369 |
| ENSG00000168274 | HIST1H2AE | 6 | 26217165 | 26217711 | 1 | protein_coding | 3012 |
| ENSG00000182952 | HMGN4 | 6 | 26538633 | 26546482 | 1 | protein_coding | 10473 |
| ENSG00000186470 | BTN3A2 | 6 | 26365387 | 26378546 | 1 | protein_coding | 11118 |
| ENSG00000187990 | HIST1H2BG | 6 | 26216428 | 26216872 | -1 | protein_coding | 8339 |
| ENSG00000196331 | HIST1H2BO | 6 | 27861203 | 27861669 | 1 | protein_coding | 8348 |
| ENSG00000197153 | HIST1H3J | 6 | 27858093 | 27860884 | -1 | protein_coding | 8356 |
| ENSG00000198327 | HIST1H4F | 6 | 26240561 | 26240976 | 1 | protein_coding | 8361 |
| ENSG00000215979 | AL021917.1 | 6 | 26365704 | 26365780 | -1 | miRNA | - |
| ENSG00000220875 | HIST1H3PS1 | 6 | 26322104 | 26322520 | -1 | pseudogene | 100289545 |
| ENSG00000228223 | HCG11 | 6 | 26522076 | 26526807 | 1 | lincRNA | 493812 |
| ENSG00000233224 | HIST1H2AM | 6 | 27860477 | 27860963 | -1 | protein_coding | 8336 |
| ENSG00000238610 | RNU7-26P | 6 | 27865282 | 27865344 | -1 | snRNA | 100147813 |
| ENSG00000252399 | RNU6-1259P | 6 | 26353199 | 26353264 | -1 | snRNA | - |
| ENSG00000261353 | CTA-14H9.5 | 6 | 26527291 | 26527632 | 1 | lincRNA | - |
| Stress-related disorder and knee OA | | | | | | | |
| ENSG00000265566 | - | - | - | - | - | - | - |

ENSG: Ensemble gene ID, Symbol: Gene Symbol, CHR: chromosome, Start: Starting position of the gene, End : Ending position of the gene, Strand : Strand of the gene, Type : Gene biotype from Ensembl

**S****upplementary Figure 1 Study flowchart for the matched cohort assessing the association of** **psychiatry disorders with subsequent osteoarthritis**

***50,543*** exposed group

***502,507*** individuals in the UK Biobank

*22040* excluded

98 withdrew from UK Biobank

6 lost to follow-up before a diagnosis of psychiatry disorders

19450 diagnosed with psychiatry disorders before 1997

2486 with a history of any osteoarthritis before the date of psychiatry disorders diagnose

*50,543* with first diagnosis of depression between January 1,1997 and December 31, 2019

480467 Eligible participants

***252,715*** matched unexposed group

1:5 individual matched by birth year (±2 year), and sex

***44,753*** exposed group

***502,507*** individuals in the UK Biobank

10682 excluded

98 withdrew from UK Biobank

4 lost to follow-up before a diagnosis of osteoarthritis

1731 diagnosis with osteoarthritis before 1997

8849 with a history of any psychiatry disorders before the date of OA diagnosis

*44,753* with first diagnosis of hip/knee osteoarthritis between January 1,1997 and December 31, 2019

*491,825* Eligible participants

***223,765*** matched unexposed group

1:5 individual matched by birth year (±2 year), and sex

**Supplementary Figure 2 Study** **flowchart for the matched cohort assessing the association of osteoarthritis with subsequent psychiatry disorders**

Eligible participants

488,377

97,059,328

Excluded participants who withdrew, or non non-white British ancestry;

Restricted to the autosomal biallelic SNPs and imputation accuracy score > 0.1

Participants with genotyping rate > 98%;

SNPs with call rate > 98%, a minor allele frequency > 0·01, and Hardy–Weinberg equilibrium (p < 10^−6^)

Participants with heterozygosity within ±3 SD from mean, without related individuals (i.e., kinship coefficients < 0.044)

**Participants and SNPs passed GWAS quality control steps**

338,573

**338,573**

7,130,905

**7,130,905**

**SNPs**

**Participants**

408,823

86,178,275

408,823

***338,573 participants included in the PRS association analysis***

49255 individuals with any psychiatry disorders

19956 with depression

19054 with anxiety

17001 with substance misuse

9368 with stress-related disorder

1028 with psychotic disorder

39,627 individuals with hip/knee osteoarthritis

16,970 with hip osteoarthritis

25,809 with knee osteoarthritis

**Supplementary Figure 3 Study flowchart for PRS analyses**

GWAS=genome-wide association studies; PRS=polygenic risk score
